# Oncologic Outcome and Efficacy of Chemotherapy in Colorectal Cancer Patients Aged 80 Years or Older

**DOI:** 10.3389/fmed.2020.525421

**Published:** 2020-10-23

**Authors:** Wenting Liu, Mengyuan Zhang, Jun Wu, Ran Tang, Liqun Hu

**Affiliations:** ^1^Geriatric Department, The First Affiliated Hospital of USTC, Division of Life Sciences and Medicine, University of Science and Technology of China (USTC), Hefei, China; ^2^Department of Pediatric Surgery, Anhui Provincial Children's Hospital, Hefei, China

**Keywords:** colorectal cancer, chemotherapy, 80 years, population-based, survival

## Abstract

**Purpose:** The present study aimed to evaluate the oncologic outcomes of patients 80 years or older compared with younger patients, and we then further investigated the efficacy of chemotherapy in individuals 80 years or older.

**Methods:** A retrospective analysis was conducted using the Surveillance, Epidemiology and End Results database. The χ^2^ test was used to analyze the different clinicopathologic and demographic variables between 65- and 79-year and ≥80-year groups. Kaplan–Meier analysis and log-rank testing were used to compare colorectal cancer (CRC)–specific survival (CCSS) curves between different groups. Multivariate and univariate Cox proportional hazards models with hazard ratios (HRs) and 95% confidence intervals (CIs) were also used to assess CCSS and OS.

**Results:** A total of 189,926 patients were included in our study. Compared with 65- to 79-year-old patients, age 80 years or older was associated with 48.4% increased CRC-specific mortality (HR = 1.484, 95% CI = 1.453–1.516, *P* < 0.0001; using 65–79 years old as the reference). Moreover, not receiving chemotherapy was significantly associated with an increased risk of CRC-related death, independent of other prognostic factors (HR = 0.615, 95% CI = 0.589–0.643, *P* < 0.0001) in individuals 80 years or older.

**Conclusions:** This large population-based study showed that older age was associated with worse oncologic outcomes compared to younger age. Chemotherapy could offer survival benefit for very old patients diagnosed with CRC, and we strongly believed that very old patients were undertreated in the present medical practices.

## Introduction

Colorectal cancer (CRC) is one of the commonest malignancies and the second commonest cancer cause of death worldwide (1). CRC is a disease that predominantly occurs in the elderly, and the incidence and mortality of CRC are expected to increase continuously with the coming of aging population (2). In the United States, the median age of patients diagnosed with CRC was 68 years. Moreover, 23.2% of new cases occurred in 75- to 84-year-olds and 12.1% in individuals older than 84 years (3). In Japan, 70% of the new cases of CRC were in people older than 65 years and 40% of those older than 75 years; the CRC mortality in these age groups had markedly increased in 2008, meaning that it was high time to conduct researches focused on this group of patients (4).

As for elderly patients diagnosed with CRC, they usually had distinct characteristics including multiple comorbidities and decreased physical functions that need to be taken account into their therapy decisions. Consequently, intensive therapies including curative surgery and adjuvant therapy were less likely to be recommended for older patients, although previous research had found that chemotherapy showed similar efficacy in elderly and younger patients (5–9). With the development of the medical techniques and the increasing of life expectancy, however, the number of patients increased greatly in the very old patients and more appropriate treatments should be carefully considered for CRC.

Moreover, that age was deemed as an independent prognostic factor in CRC had still been controversial (10–16). Therefore, we aimed to evaluate the oncologic outcomes of patients 80 years or older compared with younger patients, and we then further investigated the efficacy of chemotherapy in individuals 80 years or older.

## Materials and Methods

### Data Source

A retrospective analysis was conducted using the Surveillance, Epidemiology and End Results (SEER) database. The SEER database had combined cancer-related characteristics on the demographic, survival, and clinical information for patients diagnosed with cancer from a total of 20 cancer registries, which account for about 28% of the population of the United States.

### Study Population

We then identified a cohort of patients 65 years or older who were diagnosed with CRC between January 1, 2004, and December 31, 2015. Patients with surgical resection, positive histological confirmation, and active follow-up were identified from the SEER database.

Then, patients were excluded if (1) with unknown race, (2) with unknown T stage, (3) with unknown N stage, (4) with unknown M stage, and (5) with non-adenocarcinoma histologies. In all, a total of 189,926 patients diagnosed with CRC were included in our analyses. According to the age at diagnosis, patients were divided into two groups: <80 years and ≥80 years. Further, we also identified patients 80 years or older to evaluate the efficacy of chemotherapy in older patients, in which patients 80 years or older were further divided into chemotherapy and no chemotherapy groups. The common clinicopathological and demographic characteristics were included in our analyses (age at diagnosis, T stage, N stage, M stage, race, gender, year of diagnosis, tumor location, tumor grade, and tumor histology).

### Statistical Analyses

In our analyses, the χ^2^ test was used to analyze the different clinicopathologic and demographic variables between 65- and 79-year and ≥80-year groups. We used CRC-specific survival (CCSS) and overall survival (OS) as the endpoints of the present study. CCSS was calculated from the date of diagnosis with CRC to the date of death from CRC. Patients who died of non-CRC-related causes were censored at the date of death.

We then used Kaplan–Meier analysis and log-rank testing to compare CCSS between different groups. Multivariate and univariate Cox proportional hazards models with hazard ratios (HRs) and 95% confidence intervals (CIs) were also used to assess CCSS and OS. Two-sided *P* < 0.05 was considered statistically significant. Statistical analyses were performed using SPSS statistical software of version 22 (IBM Corporation, Armonk, NY, USA).

## Results

### Patient Characteristics

A total of 189,926 patients 65 years or older who were diagnosed with CRC were identified. The median follow-up time of the whole cohort was 41 months. Shown in Table 1, the characteristics of the eligible patients were summarized. Among all the patients, 121,358 (63.9%) of them were younger than 80 years, and 68,568 (36.1%) of them were 80 years or older. CRC patients included in our study were more likely to be T3 stage, N0 stage, M0 stage, white race, colon cancer, high/moderate tumor grade, and adenocarcinoma. It is also found from Table 1 that patients 80 years or older were more likely to be associated with higher T stage (*P* < 0.0001), N0 stage (*P* < 0.0001), M0 stage (*P* < 0.0001), white race (*P* < 0.0001), female (*P* < 0.0001), earlier years of diagnosis (*P* < 0.0001), colon cancer (*P* < 0.0001), poor/anaplastic tumor grade (*P* < 0.0001), and mucinous/signet ring cell histology (*P* < 0.0001) and less likely to receive chemotherapy (*P* < 0.0001).

**Table 1:** The results of comparison of patient characteristics.

**Variables**	**No. of patients (%)**		***P***
	**65–79 years (*n* = 121,358)**	**≥80 years (*n* = 68,568)**	
T stage			<0.0001
T1	22,382 (18.4)	8,798 (12.8)	
T2	19,861 (16.4)	11,305 (16.5)	
T3	63,552 (52.4)	38,533 (56.2)	
T4	15,563 (12.8)	9,932 (14.5)	
N stage			<0.0001
N0	75,390 (62.1)	44,321 (64.6)	
N1	28,615 (23.6)	15,214 (22.2)	
N2	17,353 (14.3)	9,033 (13.2)	
M stage			<0.0001
M0	107,415 (88.5)	62,127 (90.6)	
M1	13,943 (11.5)	6,441 (9.4)	
Race			<0.0001
White	98,717 (81.3)	59,756 (87.1)	
Black	12,852 (10.6)	4,464 (6.5)	
Other	9,789 (8.1)	4,348 (6.3)	
Gender			<0.0001
Male	64,574 (53.2)	28,471 (41.5)	
Female	56,784 (46.8)	40,097 (58.5)	
Year of diagnosis			<0.0001
2004–2007	44,515 (36.7)	25,583 (37.3)	
2008–2011	39,788 (32.8)	23,269 (33.9)	
2012–2015	37,955 (30.5)	19,716 (28.8)	
Tumor site			<0.0001
Colon cancer	93,512 (77.1)	57,743 (84.2)	
Rectal cancer	27,846 (22.9)	10,825 (15.8)	
Grade			<0.0001
High/Moderate	93,906 (77.4)	51,207 (74.7)	
Poor/Anaplastic	21,570 (17.8)	14,920 (21.8)	
Unknown	5,882 (4.8)	2,441 (3.6)	
Histotype			<0.0001
Adenocarcinoma	111,323 (91.7)	61,816 (90.2)	
Mucinous/signet ring cell	10,035 (8.3)	6,752 (9.8)	
Chemotherapy			<0.0001
No	80,359 (66.2)	60,127 (87.7)	
Yes	40,999 (33.8)	8,441 (12.3)	

### Increased Risk of CRC-Specific Mortality in Patients 80 Years or Older

Table 2 shows the results of multivariate Cox regression analyses of CCSS. T4 stage was independently associated with 375.8% increased risk of cancer-specific mortality compared with T1 stage (HR = 1.316, 95% CI = 1.243–1.394 for T2 stage; HR = 2.657, 95% CI = 2.533–2.786 for T3 stage; HR = 4.758, 95% CI = 4.520–5.009 for T4 stage; using T1 stage as the reference, *P* < 0.0001); N2 stage was independently associated with 207.6% increased risk of cancer-specific mortality compared with N0 stage (HR = 2.028, 95% CI = 1.976–2.082 for N1 stage; HR = 3.076, 95% CI = 2.989–3.165 for N2 stage; using N0 stage as the reference, *P* < 0.0001); M1 stage was independently associated with 328.6% increased risk of cancer-specific mortality compared with M0 stage (HR = 4.286, 95% CI = 4.181–4.393; using M0 stage as the reference, *P* < 0.0001); black race was independently associated with 23.0% increased risk of cancer-specific mortality compared with white race (HR = 1.230, 95% CI = 1.191–1.271 for black race; HR = 0.961, 95% CI = 0.926–0.998 for other race; using white race as the reference, *P* < 0.0001); female was independently associated with 2.6% decreased risk of cancer-specific mortality compared with male (HR = 0.974, 95% CI = 0.955–0.994; using male as the reference, *P* = 0.010); 2012–2015 was independently associated with 14.1% decreased risk of cancer-specific mortality compared with 2004–2007 (HR = 0.916, 95% CI = 0.895–0.937 for 2008–2011; HR = 0.859, 95% CI = 0.836–0.883 for 2012–2015; using 2004–2007 as the reference, *P* < 0.0001); colon cancer was independently associated with 26.9% increased risk of cancer-specific mortality compared with rectal cancer (HR = 1.269, 95% CI = 1.238–1.301; using rectal cancer as the reference, *P* < 0.0001); poor/anaplastic tumor grade was independently associated with 21.1% increased risk of cancer-specific mortality compared with high/moderate tumor grade (HR = 1.211, 95% CI = 1.184–1.239 for poor/anaplastic tumor grade; HR = 1.087, 95% CI = 1.027–1.152 for unknown tumor grade; using high/moderate tumor grade as the reference, *P* < 0.0001), whereas the difference between different histotypes did not reach statistical significance (HR = 1.012, 95% CI = 0.980–1.045; using adenocarcinoma histology, *P* = 0.482); receiving chemotherapy was independently associated with 35.6% decreased risk of cancer-specific mortality compared with not receiving chemotherapy (HR = 0.644, 95% CI = 0.629–0.660; using no chemotherapy as the reference, *P* < 0.0001). Furthermore, compared with 65- to 79-year-old patients, age 80 years or older was associated with 48.4% increased risk of CRC-specific mortality (HR = 1.484, 95% CI =1.453–1.516, *P* < 0.0001; using 65–79 years old as the reference).

**Table 2:** Results of multivariate Cox regression analyses of cancer-specific survival in the whole cohort.

**Variables**	**Cancer-specific survival**		
	**HR (95% CI)**	**SE**	***P***
Age at diagnosis			
65–79 years	Ref		
≥80 years	1.484 (1.453**–**1.516)	0.011	<0.0001
T stage			<0.0001
T1	Ref		
T2	1.316 (1.243**–**1.394)	0.029	<0.0001
T3	2.657 (2.533**–**2.786)	0.024	<0.0001
T4	4.758 (4.520**–**5.009)	0.026	<0.0001
N stage			<0.0001
N0	Ref		
N1	2.028 (1.976**–**2.082)	0.013	<0.0001
N2	3.076 (2.989**–**3.165)	0.015	<0.0001
M stage			
M0	Ref		
M1	4.286 (4.181**–**4.393)	0.013	<0.0001
Race			<0.0001
White	Ref		
Black	1.230 (1.191**–**1.271)	0.017	<0.0001
Other	0.961 (0.926**–**0.998)	0.019	0.040
Gender			
Male	Ref		
Female	0.974 (0.955**–**0.994)	0.010	0.010
Year of diagnosis			<0.0001
2004–2007	Ref		
2008–2011	0.916 (0.895**–**0.937)	0.012	<0.0001
2012–2015	0.859 (0.836**–**0.883)	0.014	<0.0001
Tumor site			
Colon cancer	Ref		
Rectal cancer	1.269 (1.238**–**1.301)	0.013	<0.0001
Grade			<0.0001
High/moderate	Ref		
Poor/anaplastic	1.211 (1.184**–**1.239)		<0.0001
Unknown	1.087 (1.027**–**1.152)		0.004
Histotype			
Adenocarcinoma	Ref		
Mucinous/signet ring cell	1.012 (0.980**–**1.045)	0.016	0.482
Chemotherapy			
No	Ref		
Yes	0.644 (0.629**–**0.660)	0.012	<0.0001

### Evaluation of the Efficacy of Chemotherapy in ≥80-Year-Old Patients

**Figure 1:**
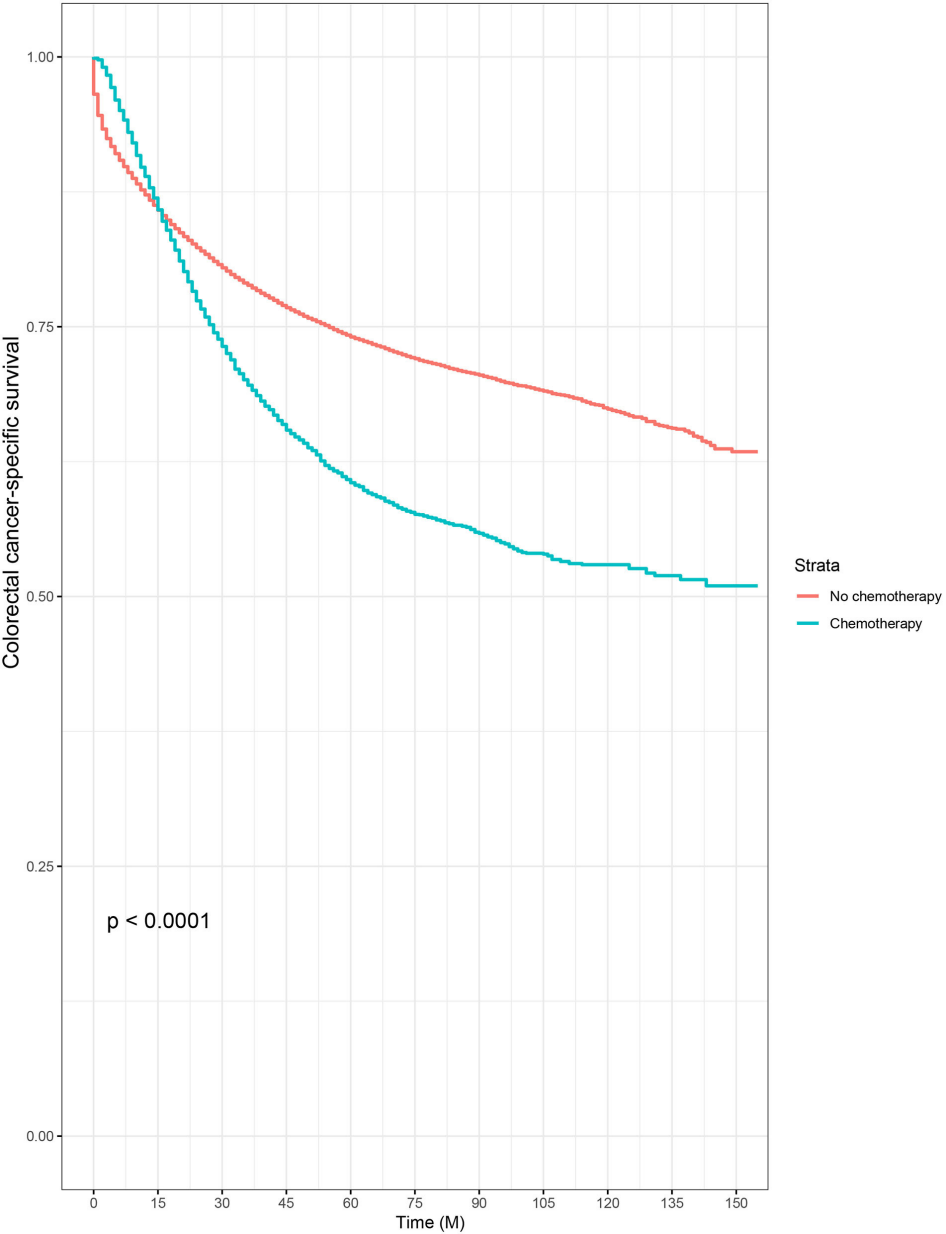
Kaplan–Meier colorectal cancer–specific survival curves based on the receipt of adjuvant chemotherapy in patients 80 years or older.

**Figure 2:**
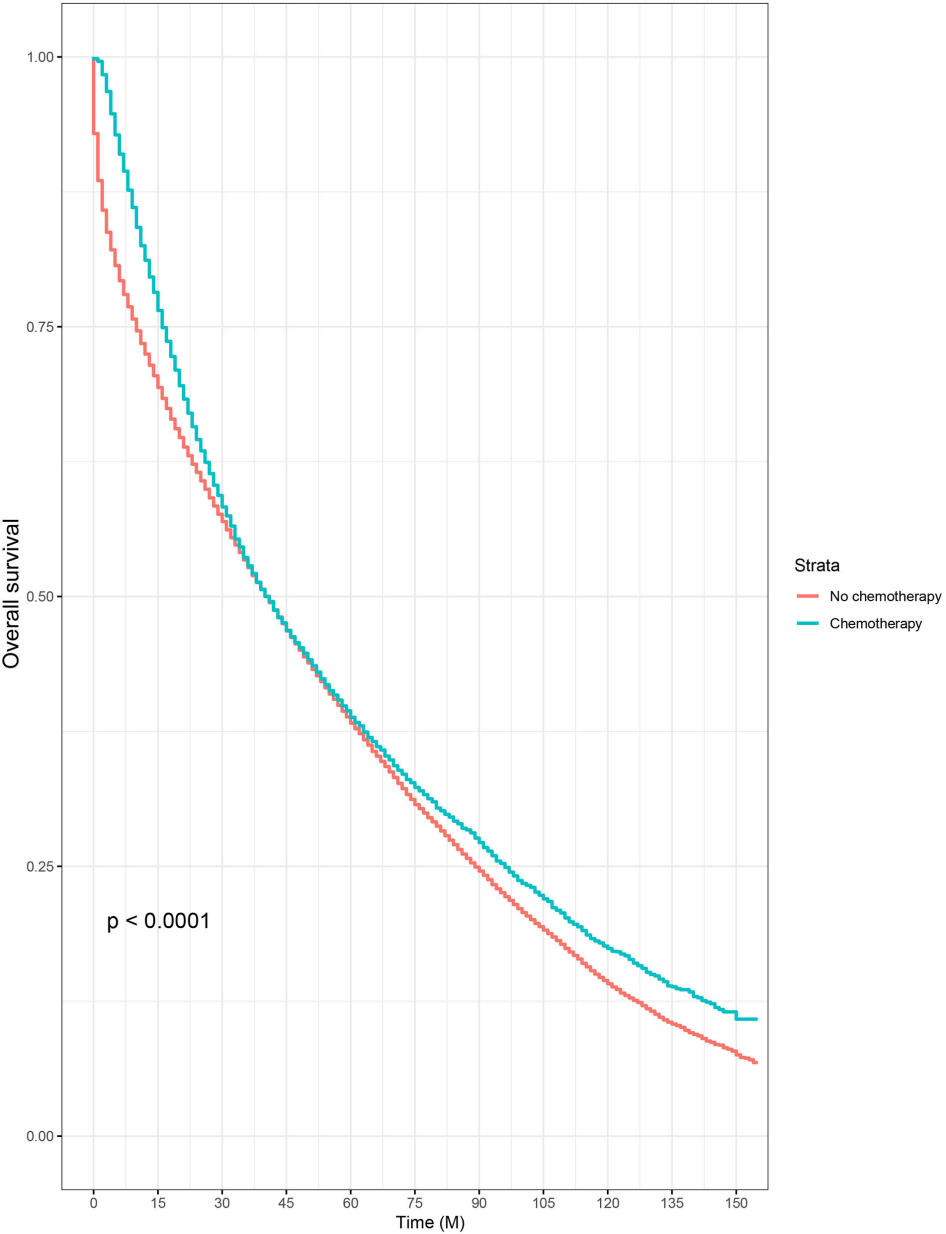
Kaplan–Meier overall survival curves based on the receipt of adjuvant chemotherapy in patients 80 years or older.

Then, we selected patients with the age of diagnosis 80 years old or more from the whole cohort. In all, 68,568 patients were included in our further analyses. In Table 3, the clinicopathological characteristics of ≥80-year-old patients are summarized. Higher T stage (*P* < 0.0001), higher N stage (*P* < 0.0001), M1 stage (*P* < 0.0001), male (*P* < 0.0001), later years of diagnosis (*P* < 0.0001), rectal cancer (*P* < 0.0001), poor tumor grade (*P* < 0.0001), and mucinous/signet ring cell (*P* = 0.006) were more likely to be associated with the receipt of chemotherapy. Kaplan–Meier CCSS and OS curves are shown in Figures 1, 2. In patients 80 years or older, the CCSS between receiving and not receiving chemotherapy was illustrated by Kaplan–Meier plots (Figure 1). It was found that receiving chemotherapy showed significantly worse CCSS compared with not receiving chemotherapy, and the 5-year CCSS rates of the two groups were 60.5 and 74.0%, respectively (*P* < 0.0001). However, not receiving chemotherapy had significantly reduced OS rate compared with receiving chemotherapy, although the two groups showed similar OS between 2.5- and 5-year survival (*P* < 0.0001, Figure 2). To further investigate the efficacy of chemotherapy in ≥80-year-old patients, we then conducted multivariate analyses using Cox proportional hazards models. In the multivariate analyses, T stage, N stage, M stage, race, gender, year of diagnosis, tumor location, tumor grade, and histotype analyzed as continuous or categorized variable were independent prognostic factors of CCSS (Table 4). Moreover, chemotherapy was demonstrated to be an independent factor for predicting CCSS, and not receiving chemotherapy was significantly associated with an increased risk of CRC-related death, independent of other prognostic factors (HR = 0.615, 95% CI = 0.589–0.643, *P* < 0.0001; Table 4). We also used multivariate Cox analyses of OS to confirm our finding that chemotherapy was demonstrated to be an independent factor for predicting OS, and not receiving chemotherapy was significantly associated with an increased risk of mortality, independent of other prognostic factors (HR = 0.538, 95% CI = 0.523–0.555, *P* < 0.0001; Table 5).

**Table 3:** The results of comparison of clinicopathologic characteristics in patients aged ≥80 years.

**Variables**	**No. of patients (%)**		***P***
	**No chemotherapy (*n* = 60,127)**	**Chemotherapy (*n* = 8,441)**	
T stage			<0.0001
T1	8,434 (14.0)	364 (4.3)	
T2	10,610 (17.6)	695 (8.2)	
T3	33,037 (54.9)	5,496 (65.1)	
T4	8,046 (13.4)	1,886 (22.3)	
N stage			<0.0001
N0	42,144 (70.1)	2,177 (25.8)	
N1	11,459 (19.1)	3,755 (44.5)	
N2	6,524 (10.9)	2,509 (29.7)	
M stage			<0.0001
M0	55,530 (92.4)	6,597 (78.2)	
M1	4,597 (7.6)	1,844 (21.8)	
Race			<0.0001
White	52,488 (87.3)	7,268 (86.1)	
Black	3,917 (6.5)	547 (6.5)	
Other	3,722 (6.2)	626 (7.4)	
Gender			<0.0001
Male	24,482 (40.7)	3,989 (47.3)	
Female	35,645 (59.3)	4,452 (52.7)	
Year of diagnosis			<0.0001
2004–2007	22,687 (37.7)	2,896 (34.3)	
2008–2011	20,374 (33.9)	2,895 (34.3)	
2012–2015	17,066 (28.4)	2,650 (31.4)	
Tumor site			<0.0001
Colon cancer	51,850 (86.2)	5,893 (69.8)	
Rectal cancer	8,277 (13.8)	2,548 (30.2)	
Grade			<0.0001
High/moderate	45,579 (75.8)	5,628 (66.7)	
Poor/anaplastic	12,458 (20.7)	2,462 (29.2)	
Unknown	2,090 (3.5)	351 (4.2)	
Histotype			0.006
Adenocarcinoma	54,277 (90.3)	7,539 (89.3)	
Mucinous/signet ring cell	5,850 (9.7)	902 (10.7)	

**Table 4:** Results of multivariate Cox regression analyses of cancer-specific survival in patients aged ≥80 years.

**Variables**	**Cancer-specific survival**		
	**HR (95% CI)**	**SE**	***P***
T stage			<0.0001
T1	Ref		
T2	1.222 (1.116–1.338)	0.046	<0.0001
T3	2.275 (2.107–2.457)	0.039	<0.0001
T4	4.089 (3.765–4.442)	0.042	<0.0001
N stage			<0.0001
N0	Ref		
N1	1.968 (1.891–2.049)	0.020	<0.0001
N2	2.993 (2.861–3.132)	0.023	<0.0001
M stage			
M0	Ref		
M1	3.576 (3.429–3.729)	0.021	<0.0001
Race			<0.0001
White	Ref		
Black	1.212 (1.143–1.286)	0.030	<0.0001
Other	1.018 (0.957–1.083)	0.032	0.565
Gender			
Male	Ref		
Female	1.040 (1.007–1.074)	0.017	0.019
Year of diagnosis			<0.0001
2004–2007	Ref		
2008–2011	0.933 (0.900–0.967)	0.018	<0.0001
2012–2015	0.886 (0.850–0.925)	0.022	<0.0001
Tumor site			
Colon cancer	Ref		
Rectal cancer	1.287 (1.232–1.344)	0.022	<0.0001
Grade			<0.0001
High/moderate	Ref		
Poor/anaplastic	1.157 (1.116–1.200)	0.018	<0.0001
Unknown	1.172 (1.065–1.290)	0.049	0.001
Histotype			
Adenocarcinoma	Ref		
Mucinous/signet ring cell	0.954 (0.907–1.004)	0.026	0.072
Chemotherapy			
No	Ref		
Yes	0.615 (0.589–0.643)	0.022	<0.0001

**Table 5:** Results of multivariate Cox regression analyses of overall survival in patients aged ≥80 years.

**Variables**	**Overall survival**		
	**HR (95% CI)**	**SE**	***P***
T stage			<0.0001
T1	Ref		
T2	1.020 (0.984–1.057)	0.018	0.278
T3	1.218 (1.181–1.256)	0.016	<0.0001
T4	1.827 (1.759–1.898)	0.019	<0.0001
N stage			<0.0001
N0	Ref		
N1	1.415 (1.382–1.448)	0.012	<0.0001
N2	1.996 (1.938–2.054)	0.015	<0.0001
M stage			
M0	Ref		
M1	2.688 (2.607–2.771)	0.016	<0.0001
Race			<0.0001
White	Ref		
Black	1.091 (1.053–1.131)	0.018	<0.0001
Other	0.821 (0.789–0.853)	0.020	<0.0001
Gender			
Male	Ref		
Female	0.840 (0.824–0.855)	0.009	<0.0001
Year of diagnosis			<0.0001
2004–2007	Ref		
2008–2011	0.981 (0.961–1.002)	0.011	0.072
2012–2015	0.923 (0.899–0.948)	0.013	<0.0001
Tumor site			
Colon cancer	Ref		
Rectal cancer	1.136 (1.108–1.165)	0.013	<0.0001
Grade			<0.0001
High/moderate	Ref		
Poor/anaplastic	1.112 (1.088–1.137)	0.011	<0.0001
Unknown	1.161 (1.105–1.220)	0.025	<0.0001
Histotype			
Adenocarcinoma	Ref		
Mucinous/signet ring cell	1.034 (1.004–1.065)	0.015	0.028
Chemotherapy			
No	Ref		
Yes	0.538 (0.523–0.555)	0.015	<0.0001

## Discussion

In this study, a total of 189,926 individuals were included in our analyses, which was the largest one focused on the oncological outcomes of patients 80 years or older to the best of our knowledge. Results of the multivariate analyses showed that age 80 years or older was associated with 48.4% increased CRC-specific mortality. In previous studies investigating survival of patients diagnosed with CRC according to age, most researchers found that old age was associated with poorer oncologic outcomes. Despite this, however, that age was deemed as an independent prognostic factor in CRC had still been controversial. In 2014, a study from China found that there was no significant difference in both disease-free survival (DFS) and OS between older and younger patients diagnosed with CRC (5). Recently, Bo et al. (17) used 80 years old as the dividing point to compare oncologic outcomes in CRC patients. And they also found that there were no differences in DFS and CSS according to age after a propensity score-matched analysis, and they believed that age was not an independent prognostic factor. Nevertheless, considering the sample sizes of the two studies were not more than 300 in ≥80-year-old groups, based on the analyses of 68,568 patients 80 years or older and other previous studies, we strongly believed that older patients were associated with worse oncologic outcomes than younger ones diagnosed with CRC.

Older individuals were often associated with multiple comorbidities and decreased physical functions; many oncologists then had the concerns that elderly patients might experience morbidity and mortality from comorbidities rather than the cancer itself, making them less likely to be offered intensive cancer treatment, and it was reported that patients younger than 80 years were three times more likely to be offered treatment than older patients (18). It was found that advanced age itself could deter doctors from choosing intensive cancer treatment, even with the fact that some patients were highly functional and lacked comorbidities, which could result into undertreatment in these older individuals diagnosed with CRC (19).

Furthermore, very old patients were underrepresented in previous clinical trials. Although ~31% of patients were diagnosed with the age of older than 75 years, only 2% of these patients were included in the studies (18, 20). As the population aged, however, the numbers of patients would be increasing in the very old group, meaning that treatment therapies of these patients should arouse much more attention of us. In the present study, it was found that very old patients (80 years old or more) with some adverse prognostic factors including higher T stage, higher N stage, M1 stage, male, poor tumor grade, and mucinous/signet ring cell histotype were more likely to receive chemotherapy, which could explain the cross between two cancer-specific survival curves that chemotherapy might be beneficial to patients' survival when <1 year, but patients without chemotherapy would have better survival outcomes than those who had chemotherapy for survival longer than 15 months in Kaplan–Meier analyses of our study. After adjusting for other prognostic factors, the results of multivariate analyses showed that chemotherapy was an independent factor for predicting CCSS, and receiving chemotherapy was significantly associated with 38.5% decreased risk of CRC-related death and 46.2% decreased risk of overall mortality, independent of other prognostic factors.

The survival benefit of adjuvant chemotherapy for CRC has not been clearly defined in very old patients. In 2001, Sargent et al. (21) found that patients aged than 70 years were associated with improved OS after receiving 5-fluorouracil (5-FU)/leucovorin or 5-FU/levamisole regimen of chemotherapy. Later in 2012, although it led to a modest increase in toxicity relative to a single 5-FU regimen, Sanoff et al. (22) reported that the addition of oxaliplatin to 5-FU was associated with better survival among patients 65 years or older. Recently, using clinical data of patients with CRC from 22 hospitals in the Japanese Study Group for Post-operative Follow-up of Colorectal Cancer, a study from Japan found that, in patients aged ≥75 years, adjuvant therapy was an independent prognostic factor and improved DFS in patients with stages II and III, disease-specific survival in patients with stage II, and OS in patients with stages II and III CRC. In our study, with the analyses of 68,568 patients older than 80 years, we had presented new data that chemotherapy could offer survival benefit (both OS and CCSS) for very old patients diagnosed with CRC. Therefore, we held the view that very old patients were undertreated under the present medical practices, and older CRC patients should be treated with chemotherapy, although further evidences need to be provided in future researches.

Our study had some inherent limitations. First, the details regarding chemotherapy were unknown (regimen, dose adjustment, completion rate, and duration). Second, the SEER database did not collect data on the tumor recurrence, RAS, BRAF status, and post-operative complications. Finally, as a retrospective study based on American instead of Chinese registries, there could be more or less confounding biases in our analyses, and prospective ones should be conducted in the future.

In conclusion, with the analyses of 189,926 patients diagnosed with CRC from SEER database, it was found that older patients were associated with worse oncologic outcomes than younger ones. Further analyses showed chemotherapy could offer survival benefit (both OS and CCSS) for very old patients diagnosed with CRC. Therefore, we held the view that very old patients were undertreated in the present medical practices.

## Data Availability Statement

Publicly available datasets were analyzed in this study. This data can be found here: https://seer.cancer.gov/.

## Ethics Statement

The data used in this study was from the publicly available SEER database and was approved by the Ethical Committee and Institutional Review Board of the First Affiliated Hospital of USTC.

## Author Contributions

WL, RT, and LH were responsible for conception, design, and the draft of the manuscript and contributed with a critical revision of the manuscript. WL, MZ, and JW performed the study selection, data extraction, and statistical analyses. All authors have read and approved the final version of the manuscript.

## Conflict of Interest

The authors declare that the research was conducted in the absence of any commercial or financial relationships that could be construed as a potential conflict of interest.
